# Aqua Walking as an Appropriate and Healthy Winter and Summer Physical Practice? An Exploratory Study

**DOI:** 10.3390/healthcare10071258

**Published:** 2022-07-05

**Authors:** Mélina Bailly, Alicia Fillon, Line Bonjean, Dominique Lucas, Catherine Kabani, Sophie Chipon, Bruno Pereira, Martine Duclos, Julien Verney, David Thivel

**Affiliations:** 1 Laboratory of Metabolic Adaptations to Exercise under Physiological and Pathological Conditions, Clermont Auvergne University, 63000 Clermont-Ferrand, France; fillonalicia@gmail.com (A.F.); line.bonjean@etu.uca.fr (L.B.); julien.verney@uca.fr (J.V.); david.thivel@uca.fr (D.T.); 2 Observatoire National de l’Activité Physique et de la Sédentarité (ONAPS), Faculté de Médecine, Université Clermont Auvergne, 63000 Clermont-Ferrand, France; mduclos@chu-clermontferrand.fr; 3 Fédération Française de la Randonnée Pédestre, 75013 Paris, France; dlucas@ffrandonnee.fr (D.L.); ckabani@ffrandonnee.fr (C.K.); schipon@ffrandonnee.fr (S.C.); 4 Alison Wave Aqua-Walking Club, 06210 Mandelieu-la-Napoule, France; 5 Délégation à la Recherche Clinique et à l’Innovation (DRCI), 63000 Clermont-Ferrand, France; bpereira@chu-clermontferrand.fr; 6 Service de Médecine du Sport et des Explorations Fonctionnelles, CHU de Clermont-Ferrand, Université Clermont Auvergne, INRA, UNH, Unité de Nutrition Humaine, CRNH Auvergne, 63000 Clermont-Ferrand, France

**Keywords:** Aqua walking, water temperature, health outcomes, quality of life

## Abstract

(1) Background: Aqua-walking in a natural environment is a health promoting physical activity that is gaining popularity and appropriate for a variety of populations, however, to date, there is little scientific evidence supporting the efficacy and safety of this activity for older adults. The objective was to propose a preliminary exploration of psychometric and metabolic responses to an acute Aqua walking session either during winter or summer in older adults Aqua walking exercisers. (2) Methods: Heart rate, body temperature, glycemia, and blood pressure were monitored in 37 (30 women, 7 men) participants aged 52 to 83 years old in two Aqua walking sessions (water at 13 °C and 18.5 °C, respectively). Anthropometry (body weight, waist, and hip circumferences), body composition, physical activity level, sedentary time, sleep quality, quality of life, physical self-perception, and perceived health and feelings on various parameters were also assessed. (3) Results: Present results revealed a greater quality of life, physical self-perception, and perceived health in aging Aqua walking exercisers compared to those found in the literature in younger populations. None of the metabolic or psychometric measurements were found to be different between classically calibrated Aqua walking sessions performed in winter compared to summer. By contrast, there was a time effect during the sessions for body temperature (*p* < 0.001), SBP (*p* = 0.17), perceived mental and physical well-being (*p* = 0.006 and *p* < 0.001, respectively), and anxiety (*p* < 0.001). Leg discomfort also showed a time effect (*p* = 0.0009) and interaction effect (*p* = 0.025). (4) Conclusion: Aqua walking appears here to be an accessible practice that can be performed all year long with a range of physical and mental benefits for older adults. Future studies should investigate the metabolic responses of Aqua walking in different populations.

## 1. Introduction

The incontestable need for an active lifestyle at any age, favoring increased physical activity and reduced sedentary behaviors, has been widely documented during the last decades based on constantly increasing scientific evidence [1,2]. Public health recommendations continue indeed to emphasize the need to engage in at least 150 min of moderate to intensive physical activities per week in adults, while limiting our time spent seated and/or in front of screens [3,4,5]. Although adopting an active lifestyle is of high importance at any age, recent evidence suggests that in adults above 50 years old, physical activity reduces the risk for many chronic and degenerative diseases most of the time associated with aging, thus improving both morbidity and mortality risks. Based on the National Health and Nutrition Examination Survey (NHANES) 1999–2018, Perez-Lasierra et al., indeed clearly showed reduced odds ratios for sarcopenia, osteoporosis, and osteoarthritis among others, in adults (>50 years old) engaged in regular physical activities [6].

Due to its thermic and mechanical properties, water-immersed exercise has been encouraged, particularly in individuals facing functional impairments [7], and such aquatic activities using the properties of water such as density, hydrostatic pressure and buoyancy [8] keep gaining in popularity. While aquatic-exercise modalities such as water-aerobic or aqua-biking are today frequently practiced in the general population, some clinical water-based rehabilitation programs have specfically been developed and found effective in patients with chronic diseases [9]. In their work, Siqueira Andrade and collaborators for instance showed that 12 weeks of both aerobic and resistance water training, performed twice a week, had the ability to improve functional capacities while maintaining the quality of life perception among 64.3 year-old women (±3.1 years) [10]. More recently, Reichert et al., also observed improved cardiorespiratory and strength fitness in response to 16 weeks of either water-based aerobic or concurrent training among older women [11]. In their quantitative review, Meredith-Jones et al., underline that regular water exercise can exert beneficial effects on cardiorespiratory fitness, strength, and body fat distribution among aging individuals and/or patients with chronic diseases, clearly calling for further well-designed studies exploring the potential effect of non-swimming water-based exercise on overall health in these populations [12].

Importantly, when it comes to immersed exercises, the water temperature needs to be considered, particularly since it has been shown to potentially induce different metabolic and energetic results [7,13]. Some laboratory-based studies have for instance compared the energetic responses to an acute cycling exercise performed either immersed in water set at 18 degrees or at 27 degrees in healthy women [14]. According to their results, exercising in moderately-cold water increases energy expenditure, oxygen consumption, and carbohydrate oxidation while reducing perceived exertion, compared to exercising at a higher temperature [14]. Interestingly, others reported a higher heart rate with no oxygen consumption and a higher perceived exertion during upper-body exercises performed at 36 °C compared to 28 °C in men aged 65 and above [15].

Although highly necessary to better understand, propose, and adapt immersed exercises to healthy individuals or patients, the studies performed so far were all conducted in controlled environments, using immersive equipment like swimming pools or aquacabins. Developed in 2005 by Thomas Wallyn, Aqua walking consists in walking along the seaside immersed up to the diaphragm, potentially using a paddle. This recent activity attracts an increasing number of adherents now reaching about 20,000 official members around the world, and offers an outdoor, nature-based alternative, whose accessibility and efficacy need to be evaluated. In that context, the present work proposes a preliminary exploration of the overall health of aging individuals who regularly participate in Aqua walking, and their psychometric and metabolic responses to an acute Aqua walking session performed in the sea either during winter (13 °C) or summer (18.5 °C) seasons. We hypothesize that Aqua walking exercisers will present a good health-related profile and that performing an acute Aqua walking session either in the winter or summer seasons will result in similar psychometric and metabolic responses.

## 2. Materials and Methods

### 2.1. Study Design

All the included participants were asked to complete several questionnaires to evaluate their physical activity level, sedentary behaviors, Physical Self-Perceptions, sleep health, Health-Related Quality of Life (HRQOL), and Perceived Health. Anthropometric measurements and body composition were also assessed. Then, the participants performed a similar 30-min training session of Aqua walking but once during wintertime (February), in water at 13 °C, and once at the beginning of September (summer season) with a sea temperature of 18.5 °C. Before, immediately after, and 30 min post-session, their body temperature, glycemia, and blood pressure were measured. The participants’ perceived temperature, fatigue, anxiety, mental and physical well-being, and leg discomfort were also assessed before, immediately after, 30 min, and 60 min post-session. Resting heart rate was evaluated in standardized conditions before each session and then heart rate was continuously monitored during the 30-min training as well as for 3 min after the session.

### 2.2. Participants

Forty adults aged 52 to 83 years old (30 women, 10 men) were recruited from the local Aqua walking club of Mandelieu-la-Napoule (France). To be included, the participants had to have been engaged in a regular (at least twice a week) practice of Aqua walking for the last two years. This project has been performed as part of the WatHealth Research Project (CPP Sud-Est VI 2019-A00353-54) and all the participants received information sheets and signed informed consent as required by the legal ethical authorities.

### 2.3. Procedures

#### 2.3.1. Aqua Walking Training Session and Heart Rate Monitoring

The participants were asked to perform the exact same classical Aqua walking session, once during wintertime (February), in water at 13 °C, and once at the beginning of September (summer season) with a sea temperature of 18.5 °C. Supervised and conducted by a certified educator, the session consisted in: (i) progressive entry into the water, with progressive warm-up exercises; (ii) 10 min of immersed walking exercises alternating between: 5 double slow paces with a high amplitude, 10 double paces at increased speed and reduced amplitude, and 5 double short paces with a low amplitude; (iii) 15 min of non-stop team walking-in-line, with an alternating line leader; (iv) 5 min of walking solo, both sideways and backwards. This exact same session was conducted on two occasions. During the entire session and up to 3 min after, the participants’ heart rate was monitored constantly using immersive heart rate monitors Polar V800 (Polar, Paris, France). Based on Karvonen’s equation, the participants’ Heart Rate Reserve (HRR) was calculated by subtracting their measured resting heart rate from the theoretical maximal heart rate (220—age). The corresponding individual percentage of HRR was then calculated during the training and the mean values were calculated every five minutes. From this, the time spent at very light, light, moderate, and vigorous intensities during the 30-min training sessions was estimated.

#### 2.3.2. Anthropometric Measurements and Body Composition

A digital scale was used to measure body mass to the nearest 0.1 kg, and barefoot standing height was assessed to the nearest 0.1 cm by using a wall-mounted stadiometer. Waist circumference was measured at a level midway between the last rib and superior iliac crest, and hip circumference was measured as the largest hips diameter. Body composition was assessed by bioelectrical impedance analysis, performed with the Tanita MC780 multi-frequency segmental body composition analyzer. This analyzer consists in a stand-alone unit onto which the subject has to step barefoot (standard mode). Information concerning the subject (age, sex, and height) was entered by the experimenter. Once body mass had been assessed by the scale, the subject had to take grips in both hands (alongside their body) during the impedance measure (hand-to-foot bioelectrical impedance analysis (BIA)). A full segmental analysis was performed in less than 20 s. Total body fat, total fat-free mass, and body water were reported by the researcher into an excel sheet for statistical treatment. The newly developed BIA analyzer has been recently validated in healthy adults [16].

#### 2.3.3. Blood Pressure and Glycemia

Systolic and diastolic blood pressures were assessed using a portable automatic wrist electronic monitor Bp-O4 (BenemedIndustry Co., HongKong) at rest as well as right before, immediately after, and 30 min post-training sessions. Glycemia was assessed based on capillary samples using a portable glucometer Accu Chek Performa (Roche Inc., Meylan, France) right before, after and, 30 min after the training sessions.

#### 2.3.4. Physical Activity and Sedentary Time

The IPAQ questionnaire (International Physical Activity Questionnaires) is a validated questionnaire used to assess the type and amount of physical activity usually performed [17]. The questionnaire is divided into four sections respectively questioning the participants’ intense activities (e.g., lifting weights, heavy work in the garden, aerobic activities such as running or cycling at high speed); moderate activities (e.g., carrying light weights, cycling at a regular speed, activity in the gym, working in the garden, prolonged physical work at home); walking; and sedentary time. Physical activity results were expressed in Metabolic Equivalent of Task (Met) per minute per week while sedentary time is expressed in minutes per week. An individual is considered inactive when the total is less than 700 Met, sufficiently active if the total is between 700 and 2519 Met, and very active when the total exceeds 2520 Met. Regarding sedentary time, a result below 180 min per day corresponds to a low sedentary time, between 180 to 420 min per day to a moderate sedentary time, while a total time above 420 min per day means that the individual is highly sedentary.

#### 2.3.5. Epworth Scale

The Epworth Sleepiness Scale (ESS) is a patient-reported outcome instrument that measures eight different situational sleep propensities that are rated retrospectively on a scale of 0–3 [18,19]. The sum of the eight situational sleep propensities provides a measure of daytime sleepiness [20]. The total score ranges from 0 (not at all likely to doze) to 24 (highly likely to doze in all eight situations) with a score below 8 corresponding to the absence of sleep deficit, a score between 9 and 14 indicating a light deficit and a score above 15 alerting for a potential need to clinically detect sleep deficit and troubles. The ESS is widely used and has been validated in healthy adults and across several sleep disorders [18,19,20].

#### 2.3.6. Health-Related Quality of Life (HRQOL) and Health Perception

The participants’ HRQOL was assessed using the SF-36 [21,22]. Based on 36 items, this questionnaire contains eight subscales: general health, physical functioning, physical role, pain, vitality, social functioning, emotional role, and mental health. A Physical Component Scale (PCS) and Mental Component Scale (MCS) can be calculated [23]. All participants were also asked to complete a short self-administered questionnaire specifically designed to explore their perception of health (“health perception scale”). Six criteria were investigated: (1) perceived physical fitness, (2) perceived ideal weight, (3) perceived healthy balanced diet, (4) perceived sleep quality, (5) perceived stress level, and (6) perceived general health. A 10-point scale from 1 (not at all) to 10 (very much) was used to assess each item. The six individual scores were computed to obtain a global score for health perception. This questionnaire has been previously validated in adults [24].

#### 2.3.7. Physical Self-Description Questionnaire (PSDQ)

Physical self-perception was measured using the French version of the short form of the PSDQ [25,26]. This questionnaire contains 40 items that measure 11 physical self-dimensions: coordination (CO), strength (ST), flexibility (FL), endurance (EN), global self-esteem (GSE), health (HE), activity (AC), body fat (BF), sport competence (SC), global physical self-concept (GPSC), and appearance (AP). A 6-point scale from 1 (false) to 6 (true) was used to assess each item. A global score for self-perceived satisfaction and for self-esteem was then computed. A score below 2 indicates a low level of positive self-perception, a score between 2 and 4 indicates a moderate self-perception while a score above 4 corresponds to a good self-perception.

#### 2.3.8. Perceived Feelings

The participants’ perception of temperature, fatigue, anxiety, mental and physical well-being, and leg discomfort were also assessed before, after, 30 min, and 60 min after the session using 150-mm visual analog scales. The questions were for fatigue: “*how tired do you feel right now*?”—0 being not tired at all and 150 being exhausted; for anxiety: “*how anxious do you feel right now*?”—0 being not anxious at all and 150 being completely anxious; for mental well-being: “*how mentally relaxed do you feel right now*?”—0 being not mentally relaxed at all and 150 being completely mentally relaxed; for physical well-being: “*how physically relaxed do you feel right now*?”—0 being not physically relaxed at all and 150 being completely physically relaxed; and for leg discomfort: “*how painful are your legs right now*?”—0 being not painful at all and 150 being really painful. The rate of perceived exertion was also evaluated at the end of the training using a 0 to 10 scale; 0 meaning no perceived exertion at all and 10 corresponding to such a difficulty that the participant had to stop.

#### 2.3.9. Statistical Analysis

Data was analyzed using the Statistica 12 software (Statsoft, TIBCO Software, Palo Alto, CA, USA). Data are expressed as means ± standard deviations and the normality of their distribution was checked using the Kolmogorov-Smirnov test. Mixed-effect models were used to compare the evolution of the data during and after the winter vs. summer sessions, taking into account participant effect as a random effect. The following fixed effects were estimated: season group (winter and summer), time (pre-session, post-session, post-session + 30 min, and post-session + 60 min), and their interaction. The normality of residuals was analyzed as aforementioned. When appropriate, logarithmic transformation was performed. Areas under the curves were also calculated using the trapezoid methods and were compared with paired Student *t*-test, or with the Wilcoxon test when the assumptions of *t*-test were not met. The level of significance was set at *p* ≤ 0.05. When omnibus *p*-value was less than 0.05, a post hoc test (Bonferroni tests) was performed to take into account multiple comparisons.

## 3. Results

### 3.1. Participants’ Characteristics

Of the initially 40 participants enrolled, 37 completed the whole study (30 women, 7 men). The participants were aged 65.1 ± 8.9 years old with a body weight of 67.1 ± 13.0 kg. Their Fat Mass Percentage was 29.6 ± 13.1% and Fat-Free Mass was 20.6 ± 8.6 kg. Waist and hip circumference were respectively 91.3 ± 11.7 and 99.6 ± 9.4 cm.

The sample shows a physical activity level of 3726 ± 2760 Mets/min/week and a sedentary time of 1427 ± 1096 min per week, with an Epworth total score of 5.6. The participants’ PSDQ score for overall health was 4.0 ± 0.9; for coordination: 4.3 ± 1.0; for physical activity: 4.8 ± 0.9; for adiposity: 3.7 ± 1.7; for physical competences: 3.7 ± 1.7; for physical appearance: 4.1 ± 0.7; for strength: 3.4 ± 0.9; for flexibility: 3.7 ± 1.1; and for endurance: 2.9 ± 1.5; leading to a total score for perceived satisfaction of 4.3 ± 0.9 and for self-esteem of 4.5 ± 1.0. The total score for self-health perception was 6.2 ± 1.3 out of 10. Regarding HRQOL, the total score for physical health was 74.9 ± 17.3, and 71.9 ± 19.1 for mental health.

### 3.2. Metabolic and Psychometric Responses to Practice Depending on the Season

**Figure 1:**
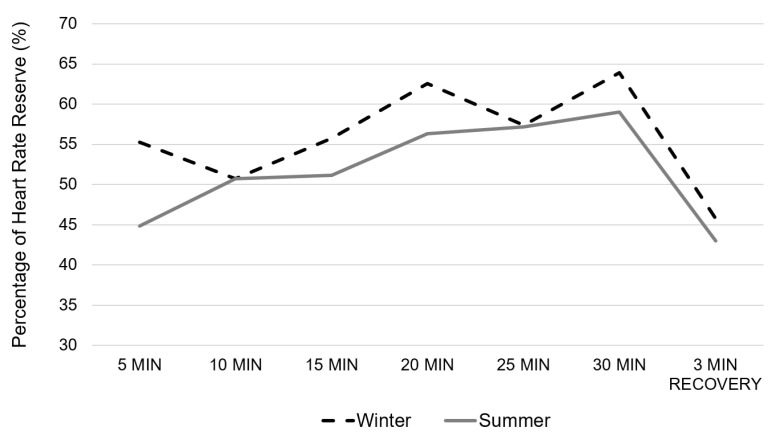
Evolution of the percentage of Heart Rate Reserve during the Aqua walking training session performed either during wintertime (dotted black line) or summertime (full grey line).

The Figure 1 presents the evaluation of the mean percentage of HRR every 5 min and after 3 min of recovery. There was no group (*p* = 0.8407) nor group × time interaction effect (*p* = 0.526); but there was however a time effect (*p* = 0.0056). The post-hoc analysis showed a significant time effect between the %HRR at 5 min compared with at 15 min (*p* = 0.0448), 20 min (*p* = 0.0032), 25 min (*p* = 0.0139) and 30 min (*p* = 0.0008); as well as between %HRR at 10 min compared with 20 min (*p* = 0.05) and 30 min (*p* = 0.0176). When considering the total Area Under the Curve (AUC), there was no difference between the two conditions (*p* = 0.5202). There was no difference between conditions regarding the %HRR after 3 min of recovery (*p* = 0.9261). The rate of perceived exertion was significantly higher during the summer condition (3.6 ± 1.9) compared to the winter one (3.2 ± 1.9) (*p* = 0.0048).

**Figure 2:**
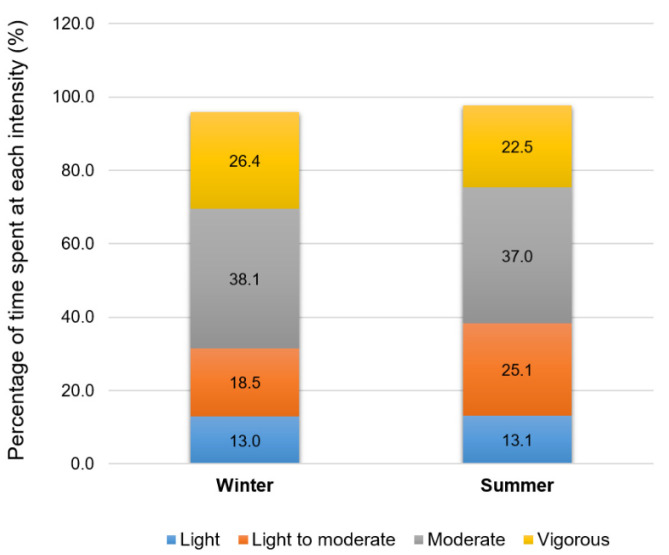
Time spent at light, light to moderate, moderate, or vigorous intensities during the Aqua walking training session performed either during wintertime or summertime.

Figure 2 illustrates the time spent at each intensity according to the American College of Sports Medicine’s (ACSM) cut-offs using the %HRR. The time spent at a light (*p* = 0.9676), light to moderate (*p* = 0.2973), moderate (*p* = 0.8946), and vigorous intensities (*p* = 0.6846) were not significantly different between seasonal conditions.

Table 1 details the results regarding the evolution of some metabolic and psychometric indicators during the winter and summer training sessions. None of the studied parameters showed any season effect (Table 1), and a season × time interaction was observed only for the self-declared leg discomfort (*p* = 0.025). While perceived fatigue, perceived temperature, diastolic blood pressure, and glycemia did not show significant time effect, body temperature (*p* < 0.0001), systolic blood pressure (*p* = 0.017), perceived mental well-being (*p* = 0.006), perceived physical well-being (*p* < 0.0001), perceived anxiety (*p* < 0.0001) and perceived leg discomfort (*p* = 0.0009) showed a general time effect. The general time effect for body temperature showed significant differences between pre and post training session values (*p* < 0.0001), and between pre and 30 min post values (*p* = 0.006), and between post and 30 min post values (*p* < 0.0001). Systolic blood pressure was found to be significantly different between pre and post training values (*p* = 0.0416), and between pre and 30 min post values (*p* = 0.002), as well as between 30 and 60 min post training (*p* = 0.026). Perceived mental well-being was found to be significantly different between pre and: post (*p* = 0.0056), 30 min post (*p* = 0.0081), and 60 min post training (*p* = 0.0022). The perceived physical well-being was found to be significantly different between pre values and: post (*p* = 0.0005), 30 min post (*p* = 0.0005), and 60 min post session values (*p* < 0.0001).

**Figure 3:**
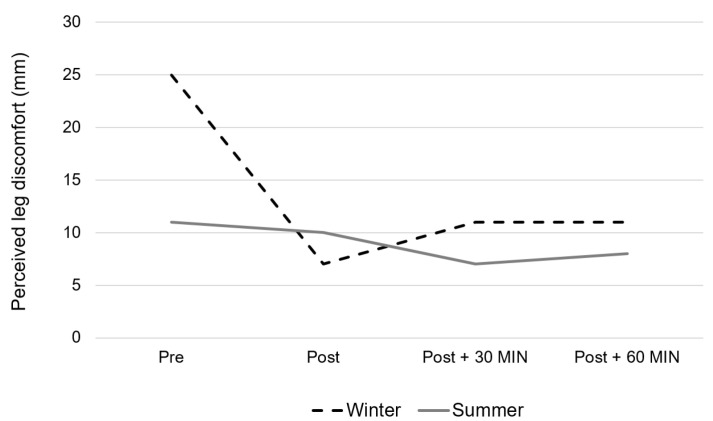
Evolution of the perceived leg discomfort during the Aqua walking training session performed either during wintertime (dotted black line) or summertime (full grey line). (Season effect: *p* = 0.46, Time effect: *p* = 0.0009, Interaction: *p* = 0.025.)

Finally, as shown in Figure 3, leg discomfort presents significant differences between pre values and: post (*p* = 0.0032), 30 min post (*p* = 0.0028), and 60 min post training values (*p* = 0.0024).

## 4. Discussion

Just as public health authorities and recommendations keep advocating for the adoption of a healthier lifestyle including increased physical activity levels and reduced sedentary time, so are aquatic and water-based activities gaining in popularity at all ages [27,28]. In addition to aquatic exercise activities performed in swimming pools or using specific equipment such as aqua-cabins, new outdoor activities performed in natural settings are being developed and proposed to the general population as well as to aging people and individuals with chronic diseases. Very recently, Ivaniski-Mello and colleagues elegantly showed, using a systematic and meta-analytic approach, that shallow water walking while immersed at the xiphoid and waist level might be an adequate activity to increase energy expenditure and cardiovascular demand, while reducing the lower limb impact force compared with dry land walking [29]. Issued from a training strategy proposed to kayak athletes, Aqua walking was developed in 2005 by Thomas Wallyn, consisting in walking along the seaside immersed up to the diaphragm, potentially using a paddle, and performing specific walking exercises. Rapidly gaining in popularity in Europe and around the world, Aqua walking is today suggested as an adapted and health-beneficial physical practice at any age in both healthy individuals and patients. The prescription of physical activities for health is today vigorously promoted and encouraged [30], and the present work proposes a preliminary evaluation of the overall health of regular Aqua walking exercisers belonging to an aging group of individuals, and their psychometric and metabolic responses to an acute Aqua walking session—performed either during winter (13 °C) or summer (18.5 °C) seasons.

According to our results, the participants of the present study are considered to be active with a moderate level of sedentary time according to their IPAQ scores, without any sleep issues (Epworth score below 8 (5.6 ± 3)) and an overall good self-perception (scored 6.2 ± 1.3 out of 10). Similarly, using the PSDQ, our results indicate that our sample shows a moderate to great perception of their overall physical coordination, physical activity, adiposity, physical competence, appearance, strength, flexibility, and endurance, positioning them with great overall self-physical satisfaction and esteem. Finally, the HRQOL evaluation reveals a great to excellent overall quality of life with sub-scores for mental and physical scores of 74.9 ± 17.3 and 71.9 ± 19.1, respectively. Interestingly, the results suggest a higher overall quality of life among our participants when compared to aged-matched non-exercisers (main physical score of 58 ± 4.6), and similar to what can be observed in regular Nordic walkers aged 65–74 years old (main physical score of 72.1 ± 4.2) [31]. In addition, while the total health self-perception of our participants is found to be similar to what was previously observed among 50 to 65 year-old postmenopausal brisk-walking women [24], it appears to be significantly higher than what has been recently shown among tertiary men and women workers aged 45–55 years old, who reported an overall health perception score between 4.2 and 5.8 out of 10 [32]. 

Although self-reported, these results tend to indicate that regular Aqua walking exercisers present a clearly great quality of life and perception of their physical and mental capacities and health when compared to younger or aged-matched active or inactive individuals. Interestingly, it also suggests that Aqua walking might have a greater effect on the quality of life and health perception in aging people compared to a land-based walking practice such as Nordic Walking. These results are in line with several previously published results highlighting a similar-to-greater impact of regular aquatic-based immersed physical activities on both functional and psychological health and well-being in aging individuals [9,10,11].

Although exercising in a natural outdoor setting certainly contributes to these positive impacts of Aqua walking on overall health, as recently suggested [33,34], such a practice might be affected by the meteorological variations between seasons and notably by the temperature of the water. Indeed, contrary to indoor pools and cabins, ambient and water temperatures, as well as the water flow, cannot be controlled when practicing in a natural seaside environment. Thus, this study also contributes to evaluating some metabolic and psychometric responses to a calibrated Aqua walking training session performed in the natural seaside setting either in winter or summer, with uncontrolled water temperatures of 13 and 18.5 °C respectively. 

According to our results, none of the metabolic, psychometric or perceptual variables under study showed significantly different responses to a classically calibrated Aqua walking session performed either during winter or summer. Only general time effects were observed for some parameters such as body temperature that slightly significantly decreased from pre to post session, going back to baseline values 30 min post session. Similarly, diastolic blood pressure was found to significantly decrease during the session, remaining lower than baseline 30 min afterwards. According to our results, performing an Aqua walking session, whatever the temperature of the water and the season, significantly improves self-reported physical and mental well-being, while reducing anxiety and leg discomfort. These results, although self-reported, suggest that such an aquatic-based physical activity provides short-term beneficial effects among aging individuals, being adapted and appropriate to both winter and summer. Moreover, by continuously monitoring the participants’ heart rate, we found that the cardio-metabolic load was not different between a session performed at 13 or 18.5 °C given the similar time effect observed. Importantly, although the rate of perceived exertion was found to be significantly higher in summer compared to winter, both remained below a score of 4 out of 10, clearly underlying the high level of acceptability among such aging people.

Interestingly, while public health recommendations for physical activity advocate for the realization of moderate to vigorous activities, our results indicate that older adults spend more than 50% of a 30-min Aqua walking session at moderate to vigorous intensities without any perceived or cardio-metabolic difficulties, regardless of the season of practice. Indeed, the participants spent about 19.3 min and 16.8 min at moderate to high intensity during the winter and summer sessions, respectively (without any difference between seasons). These results, in addition to the psychometric ones, suggest that Aqua walking is an accessible, well-adapted and health-beneficial exercise modality to be practiced all year long in older adults. It also seems pertinent to underline the very important enthusiasm of the Aqua walking exercisers for their own practice noted during the realization of the study, in line with these psychometric results.

Although this work remains exploratory, some limitations have to be considered. First, the presence of a control group, composed of age-matched individuals not engaged in regular Aqua walking, could have been of interest. Similarly, the relatively reduced size and homogeneous nature of the sample group might limit our results and their generalizability to the overall population. The evaluation of the participants’ physical activity level and sedentary time using objective tools such as accelerometers could also have been relevant, but was not feasible in the present work for practical reasons. The use of self-reported questionnaires might indeed explain the high level of physical activity observed and the relatively modest time spent in sedentary activities. Similarly, the estimation of the exercise intensity zones during the Aqua walking sessions relied on the estimation of the participants’ maximal heart rate which remains unprecise. A direct laboratory-based measure of the participants’ maximal aerobic capacities and then maximal heart rate would have been more appropriate for better detection of these intensity zones. Finally, a better repartition of the number of men and women would have increased the applicability of the study, and would have made it possible to investigate a potential and highly interesting gender effect. In summary, although this work suffers from several practical and methodological limitations, it provides some first preliminary results regarding the regular practice of Aqua walking as an interesting physical activity for health in older adults.

## 5. Conclusions

To conclude, the present preliminary work suggests Aqua walking to be an adapted and beneficial physical practice for aging individuals with various functional and metabolic profiles that can be practiced all year long. Further well-designed and better controlled studies are now needed to study the potential role of Aqua walking in the general population, considering the socio-economic, cultural, and ethnic origins of individuals, as well as in specific populations, as a potential rehabilitation alternative.

## Figures and Tables

**Table 1:** Evolution of the metabolic and psychometric variables during the Aqua walking session performed in winter and summer.

	Winter Session								Summer Session										
	Pre-Session		Post-Session		Post-Session + 30 Min		Post-Session + 60 Min		Pre-Session		Post-Session		Post-Session + 30 Min		Post-Session + 60 Min		Season	Time	Interaction
	*Mean*	*SD*	*Mean*	*SD*	*Mean*	*SD*	*Mean*	*SD*	*Mean*	*SD*	*Mean*	*SD*	*Mean*	*SD*	*Mean*	*SD*	*p-Value*	*p-Value*	*p-Value*
**Body Temperature (°C)**	36.6	0.2	36.3	0.4	36.4	0.2	/	/	36.5	0.3	36.1	0.6	36.3	0.3	/	/	0.303	<0.0001	0.805
**Glycemia (g/l)**	0.96	0.03	0.83	0.04	0.98	0.3	/	/	0.99	0.5	0.97	0.1	0.96	0.1	/	/	0.139	0.085	0.230
**Systolic Blood Pressure (mmHG)**	138	16	134	13	131	16	/	/	141	23	129	17	123	29	/	/	0.532	0.017	0.422
**Diastolic Blood Pressure (mmHG)**	86	7	85	6	85	8	/	/	94	12	86	11	84	20	/	/	0.379	0.120	0.265
**Perceived cold/hot (mm)**	87	28	90	39	90	31	74	37	70	47	60	24	65	30	45	28	0.197	0.476	0.961
**Perceived Mental well-being (mm)**	116	45	131	35	132	36	138	23	126	36	140	14	138	19	138	22	0.279	0.006	0.805
**Perceived Physical well-being (mm)**	110	42	132	35	131	36	139	18	119	35	133	26	135	23	135	27	0.675	<0.0001	0.642
**Perceived Anxiety (mm)**	31	25	29	26	18	19	19	12	33	27	27	15	20	12	15	19	0.569	<0.0001	0.215
**Perceived Fatigue (mm)**	37	40	23	36	36	48	33	41	31	31	27	36	18	25	21	32	0.292	0.337	0.163
**Perceived Leg discomfort (mm)**	25	37	7	20	11	29	11	27	11	19	10	20	7	11	8	13	0.460	0.0009	0.025

SD: Standard Deviations.

## Data Availability

Data can be sent by the authors on request.
